# Book Review: Antibody-Drug Conjugates: Fundamentals, Drug Development, and Clinical Outcomes to Target Cancer

**DOI:** 10.3389/fphar.2017.00771

**Published:** 2017-10-25

**Authors:** Dipesh Dhakal, Yogesh Dhakal, Jae K. Sohng

**Affiliations:** ^1^Department of Life Science and Biochemical Engineering, Sun Moon University, Chungnam, South Korea; ^2^Department of Anaesthesiology, Nepalgunj Medical College, Kohalpur, Nepal; ^3^Department of BT-Convergent Pharmaceutical Engineering, Sun Moon University, Chungnam, South Korea

**Keywords:** antibody drug conjugate, anticancer drugs, drug development

Antibody drug conjugate (ADC) consists of a potent cytotoxic agent payload, linked covalently to side chains of particular amino acid residues of a monoclonal antibody (mAb). Most of the cytotoxic agent including the major payload precursors used in ADC are derived from microbial sources (Dhakal et al., 2017; Wang et al., 2017). Hence, ADCs utilize a coalition of biology and chemistry for advanced drug delivery system, in which mAb guides chemically linked cytotoxic drug to target cells, where drug molecule exhibits its specific mode of action (Dhakal and Sohng, 2017; Wang et al., 2017). Thus, an ADC acts as a prodrug that is usually only activated within tumor cells.

The book (Olivier Jr and Hurvitz, 2016) is divided into distinct 5 parts with 18 chapters providing in-depth knowledge about ADCs, their recent developments, and prospects for application in cancer immunotherapy. This book provides a comprehensive overview of aspects as: (i) ADC fundamentals, (ii) chemistry and biology behind synthesis of ADCs, (iii) clinical and non-clinical aspects of ADC developments, and (iv) regulatory approval strategies for ADCs. The detailed insights on each fundamental aspect along with subsequent examples/case studies provide readers with practical and proven solutions for designing, developing, and improving the cancer immunotherapy based on ADCs. Hence, the book is a worthy resource for academic oncologists, drug researchers, and clinical developers and practitioners for attaining all dimensions of newest cutting-edge information in the field of oncology drug development, particularly focused on effective ADCs.

Part 1, chapter 1 contains fundamentals about components and characteristics of a typical ADC. Part 2 explains engineering, manufacturing, and optimization of ADC. Chapter 2 deals with required features of ideal ADC (maximal efficacy, precise specificity, and minimal toxicity). However, it is almost impossible to get antigen targets that are only expressed in tumors cells. Similarly, there are rare chances for complete localization of ADC to lysozyme following internalization, so rational optimizations of ADCs are required. Chapter 3 provides an overview on special consideration during development and characterization of ADC in terms of their quality, safety, purity, and stability. Chapter 4 focuses on linker and conjugation technologies that allow the ADC to retain better pharmacokinetic (PK) properties. Linkers are either non-cleavable (always attached to a drug metabolite) or cleavable (that release the putative drug upon cleavage inside the cellular environment). Alteration of linking chemistry by engineering THIOMABs, and site-specific coupling has been routinely utilized for improvising the PK profile of ADCs. Chapter 5 contains information on strategies and considerations for maintaining the physical and chemical stability of ADC during manufacture. The chapter also includes specific consideration during formulation and logistical considerations for maintaining consistent quality. Chapter 6 presents the specific quality control (QC) assays for characterization of ADC before the initiation of non-clinical and clinical studies. These QC assays are cell-based cytotoxicity assays, immunogenicity assays etc., that need to be completely validated prior to commercialization. Chapter 7 explains the occupational hazard associated with components of ADC. The general guidance for material handling, information on specific facility requirements and engineering control, and personal protective measures for safe manufacturing process are also explained in details.

The development of ADCs is fundamentally reliant on proper characterization and optimization of ADC before clinical use. Part 3 focuses on non-clinical aspects of designing and developments of ADC. Chapter 8 present bioanalytical strategies enabling successful ADC translation from initial designing. The bioanalysis of different components of ADC assists on assessment of integrity, bio-distribution, and PK properties, which correlates to efficacy and safety on patients. As explained in Chapter 9, in order to understand the factors modulating the efficiency of ADC, varieties of experimental models such as: *in vitro* cellular assays, or *in vivo* tumor xenograft mouse models are used. Chapter 10 explains the basic considerations for optimizing PK properties of ADCs using bioanalytical tools, *in vitro* assays, *in vivo* studies, and mathematical models. Chapter 11 provides the information about regulations for designing, formulating, evaluating and marketing of ADCs, particularly focusing on criteria for approval from Food and Drug Administration (FDA).

Part 4 deals with the clinical aspects of ADC and their development as precise therapeutics. Considerations for evaluation of ADCs from phase I and beyond; and list of ADCs that has been already approved for clinical use or in phase trials are provided in Chapter 12. Chapter 13 presents the specific consideration while designing and developing the components of such ADC. The assessment techniques for validating specific requirements for each component of ADC *viz* antibody (high specificity to target antigen), payload (inhibition of tumor proliferation), and linkers (stability for delivery and efficiency for release) are explained. Chapter 14 presents information about clinical aspects of trastuzumab emtansine (Kadcyla®, T-DM1), an ADC for the treatment of HER2-positive metastatic breast cancer. Similarly, Chapter 15 presents the clinical aspects of brentuximab vedotin (BV), an ADC with efficacy in CD30-positive malignancies.

Part 5 provides insight on future perspectives in ADC development by overcoming the present shortcomings. The efficiencies of ADCs are compromised by poor intake, rapid clearance and non-specific action on normal cells. Chapter 17 proposes that the labeled antibodies can be utilized for assessing the specificity, distribution, and dosage effect of particular antibody at specific tumor location. Chapter 18 illustrates the newer opportunities provided by additional cytotoxic drug classes with novel cellular targets, development in linker repertoire and antibody engineering. These advances in bioengineering, linker chemistry and potent cytotoxic payload have made ADC technology a powerful tool for targeted cancer therapy (Sau et al., 2017). For example utilizing biorthogonal chemistry and protein engineering, THIOMAB-drug conjugate (TDC) are generated. TDCs possess homogenous DAR (Drug-antibody ratio), low hydrophobicity, higher clinical efficacy and better patient safety for cancer therapy (Agarwal and Bertozzi, 2015). The current focus of research is on areas as: (i) development of bispecific ADCs with enhanced potency/uptake, (ii) ADCs containing multiple classes of cytotoxic payloads, and (iii) ADC with improved safety using humanized antibodies. Collectively, these advances will certainly enhance and expand the utilities of ADCs as novel targeted immunotherapies.

## Author contributions

All authors listed have made a substantial, direct and intellectual contribution to the work, and approved it for publication.

### Conflict of interest statement

The authors declare that the research was conducted in the absence of any commercial or financial relationships that could be construed as a potential conflict of interest.
